# A Functional Variant of CXCL16 Is Associated With Predisposition to Sepsis and MODS in Trauma Patients: Genetic Association Studies

**DOI:** 10.3389/fgene.2021.720313

**Published:** 2021-09-03

**Authors:** Jianhui Sun, Huacai Zhang, Di Liu, Li Cui, Qiang Wang, Lebin Gan, Dalin Wen, Jun Wang, Juan Du, Hong Huang, Anqiang Zhang, Jin Deng, Jianxin Jiang, Ling Zeng

**Affiliations:** ^1^Department of Trauma Medical Center, Daping Hospital, Army Medical University, Chongqing, China; ^2^Department of Emergency, The Affiliated Hospital of Guizhou Medical University, Guiyang, China

**Keywords:** sepsis, CXCL16, single nucleotide polymorphisms, trauma, multiple organ dysfunction

## Abstract

**Purpose:**

CXC chemokines are mediators which mediate immune cells migration to sites of inflammation and injury. Chemokine C-X-C motif ligand 16 (CXCL16) plays an important role in the occurrence and development of sepsis through leukocyte chemotaxis, leukocyte adhesion and endotoxin clearance. In this study, we selected a set of tagging single nucleotide polymorphisms (tag SNPs) in the CXCL16 gene and investigated their clinical relevance to the development of sepsis and multiple organ dysfunction syndrome (MODS) in patients with major trauma in three independent Chinese Han populations.

**Methods:**

A total of 1,620 major trauma patients were enrolled in this study. Among these patients, 920 came from Chongqing in western China, 350 came from Zhejiang Province in eastern China, and 350 came from Guizhou Province in southwestern China. The improved multiplex ligation detection reaction (iMLDR) method was employed in the genotyping and genetic association analyses to determine the associations between CXCL16 haplotypes and sepsis morbidity rate and higher MOD scores in three cohorts.

**Results:**

Only CXCL16 T123V181 haplotype was associated with an increased risk for sepsis morbidity and higher MOD scores in the three cohorts (OR = 1.89, *P* = 0.001 for the Chongqing cohort; OR = 1.76, *P* = 0.004 for the Zhejiang cohort; OR = 1.55, *P* = 0.012 for the Guizhou cohort). The effect of T123V181 haplotype on the chemotaxis, migration and endotoxin clearance of immune cells were further observed. Protein modeling analysis showed that T123 and V181 might alter the structure of the CXCL16 active center. Thus it enhanced the chemotaxis and adhesion ability of immunocytes.

**Conclusion:**

We demonstrate the mechanism of CXCL16 T123V181 haplotype which regulates the sepsis morbidity rate and thus provide a new biomarker for early diagnosis of sepsis and MODS.

**Clinical Trial Registration:**

www.ClinicalTrials.gov, identifier NCT01713205 (https://www.clinicaltrials.gov/ct2/results?cond=&term=+NCT01713205&cntry=&state=&city=&dist=).

## Introduction

With the continuous improvement of emergency and trauma care technology, the mortality of severe trauma patients caused by trauma itself has been significantly reduced. However, trauma patients often die of posttraumatic complications, including sepsis and multiple organ dysfunction syndrome (MODS) (Dellinger et al., 2013). Sepsis represents a considerable threat to human health, affects the quality of human life and has a large financial cost. Approximately 14,000 people die of sepsis and MODS every day worldwide, and this number is increasing at a rate of 1.5–8.0% every year. According to an epidemiological survey, the mortality of sepsis has exceeded that of myocardial infarction, which has become the leading cause of death in intensive care units (Seymour et al., 2017; Prescott and Angus, 2018). In recent years, the mortality of sepsis has remained as high as 30–70%, despite the considerable advances made in anti-infection treatment and organ function support technology (Fleischmann et al., 2016). Sepsis is a multifactorial disease that is regulated by pathogenic microorganisms and genetic factors. Significant differences in susceptibility and prognosis to sepsis were observed among individuals. Sørensen et al. (1988) found that genetic factors played a more important role in the prognosis of infectious diseases than in common diseases, such as cancer and cardiovascular diseases. Individuals whose parents died of infectious diseases are 5.8 times more likely to die after infection (Sørensen et al., 1988). There were significant differences in cytokine production, bacterial translocation, prognosis and mortality among mice with different gene backgrounds (Gu and Jiang, 2010). Stüber et al. (1996) first reported that polymorphisms of the TNF gene are significantly related to the susceptibility and prognosis of sepsis and organ dysfunction. Since that study was reported, extensive research has identified that genetic factors play important roles in the pathogenesis of complications after trauma. Our research group has taken the lead in conducting association studies and functional studies between genomic polymorphisms and the risk of major trauma complications in China. We sequenced the genomes of 27 Han individuals and obtained information on their single nucleotide polymorphisms (SNPs) in sepsis-related genes. Next, through association studies, we identified a series of SNPs that are closely related to the prognosis of sepsis and MODS. These SNPs are primarily located on pattern recognition receptor genes (Zeng et al., 2012a,b) and cytokine genes (Zeng et al., 2009, 2011; Gu et al., 2010), which are closely related to the pathophysiological process of trauma complications.

Large number of neutrophils are produced in bone marrow during infection, and they exert an anti-infection effect by reaching the infected site through the circulatory system (McDonald et al., 2012; Allam et al., 2014). CXC gene family members are the key component of the host defense response, serving to guide neutrophil migration to bacterial infection sites (Paudel et al., 2019). Therefore, CXC chemokine family genes play important roles in inflammatory and immune-related diseases. However, how CXC family members regulate granulogenesis, neutrophil recruitment, and neutrophil mobilization in response to sepsis caused by infection has not been elucidated (Zhang et al., 2010; Satake et al., 2012). During sepsis, alveolar macrophages are implicated in polymorphonuclear leukocyte recruitment to the lungs. Wang et al. (2008) elucidated that the chemokine C-X-C in septic plasma is responsible for the activation of alveolar macrophages. Chemokine C-X-C motif ligand 16 (CXCL16) belongs to the CXC family. CXCL16 is expressed in soluble or transmembrane forms and can be observed in many cell types, including inflammatory cells (such as macrophages, neutrophils, dendritic cells and monocytes) and non-inflammatory cells (such as lung epithelial cells and renal cells). CXCL16 has the characteristics of both CC family and CX3C family chemokines. CXCL16 is primarily expressed on the surface of antigen-presenting cells (APCs) and consists of a chemokine domain (∼89 amino acids), a mucin-type stalk (∼110 amino acids), a single-pass transmembrane domain (∼20 amino acids), and a cytoplasmic tail (∼27 amino acids) (Shimaoka et al., 2000; Wuttge et al., 2004). CXCL16 is the only ligand of the CXCR6 receptor. Soluble CXCL16 induces the migration of CXCR6+ cells (including Th1 cells, NK cells and activated CD8 + T-cells) (Matloubian et al., 2000; Wilbanks et al., 2001), M2-macrophage infiltration (Kim et al., 2019), interactions between APC and CD8 + T-cells (Yamauchi et al., 2004), the cellular immune response and inflammatory response (Carossino et al., 2019), and the development of thymocytes (Lepennetier et al., 2019). Membrane-bound CXCL16 can promote the adhesion of CXCR6+ cells (Tohyama et al., 2007; Borst et al., 2014). CXCL16 plays important roles both in the natural immune barrier and in the occurrence and development of autoimmune diseases. Fahy et al. (2006) established a model of salmonellae-induced small enteritis in mice to study the mechanism of the CXCL16 immune response *in vivo*. These researchers showed that CXCL16 induced the expression of interferon through the primary immune response to bacterial infection, thereby playing an important role in small enteritis infection regulation. A study showed that CXCL16 on dendritic cells and macrophages regulates bacterial phagocytosis and adhesion of *Staphylococcus aureus* and *Escherichia coli*. CXCL16 mediates bacterial recognition, which suggests that CXCL16 is an important chemokine in infectious diseases (Shimaoka et al., 2003).

Xu et al. (2017) observed that the CXCL16 haplotype rs2304973T-rs1050998C-rs3744700G-rs8123A significantly elevated myocardial infarction risk. A clinical study indicated that the CXCL16 missense allele haplotype T123V181 was significantly associated with carotid plaque, which may be caused by the impact of CXCL16 protein sequence variation on the interactions between CXCL16 and CXCR6 (Zivković et al., 2015).

We selected a set of tag SNPs within the entire CXCL16 gene and investigated their clinical relevance in relation to the development of sepsis and MODS in patients with major trauma in three independent Chinese Han populations. In this report, we present experimental evidence for the impact of two missense tag SNPs of CXCL16 on sepsis and MODS. Furthermore, we investigated the mechanism by which the CXCL16 genotype T123V181 promotes neutrophil chemotaxis and adhesion. Our findings may help to establish a new strategy for the early warning and diagnosis of severe trauma complications.

## Materials and Methods

### Research Populations

A total of 1,620 major trauma patients were enrolled in this study, all of whom were Han Chinese people from Chongqing in western China (*n* = 920), Zhejiang Province in eastern China (*n* = 350) and Guizhou Province in southwestern China (*n* = 350). Patients were admitted to Daping Hospital of Army Medical University, Chongqing Emergency Medical Center, The Second Affiliated Hospital of Zhejiang University and the Affiliated Hospital of Guizhou Medical University between 2005 and 2019. The inclusion criteria were as follows: (1) age between 18 and 65, (2) injury severity score (ISS) greater than 16, and (3) survival for more than 2 days after injury. The exclusion criteria were as follows: (1) combined with penetrating injuries and (2) severe brain injury or preexisting cardiovascular, respiratory, renal, hepatic, hematological or immunological diseases. Ethics approval for this study was obtained from the Ethics and Protocol Review Committees of Army Medical University, Chongqing Emergency Medical Center, Zhejiang University and Guizhou Medical University (Trial registration: ClinicalTrials.gov, NCT01713205. Registered on 18th October 2012, retrospectively registered). Before enrollment, written informed consent was obtained from the patients or their next of kin, which covered the collection of relevant clinical data and explicit DNA analysis. Patient confidentiality was preserved according to the guidelines of the Declaration of Helsinki.

### Sepsis and MODS Evaluation

Sepsis is defined as an acute change in total sequential (sepsis-related) organ failure assessment (SOFA) score greater than or equal to 2 points consequent to an infection (Singer et al., 2016). MOD scores are evaluated on each hospitalization day. Briefly, pulmonary, renal, hepatic, neurological, cardiac and hematological parameters were scored from 0 to 4 every day. The MOD scores ranged from 0 to 4, and the total score ranged from 0 to 24 (six organs). Failure of organ function was considered to be 3 or more points for more than 2 consecutive days (Marshall et al., 1995). The presence of sepsis and multiple organ dysfunction scores were determined by individuals who did not know the patients’ genotypes.

### Genotyping

Genomic DNA was isolated from peripheral whole blood by QuickGene-610L (Fujifilm, Tokyo, Japan). The concentration and purity of DNA samples were checked by a Thermo Scientific NanoDrop ND-1000 spectrophotometer (Isogen Life Science, De Meern, Netherlands). DNA samples were stored at –80°C. SNP genotyping was performed by an improved multiplex ligation detection reaction (iMLDR) technique, as described in our previous report (Lu et al., 2019). Approximately 10% of the samples were genotyped in duplicate to assess the accuracy of iMLDR. Genotyping was performed by researchers who did not know the patients’ clinical data.

### Expression Plasmid Construction

A plasmid containing human CXCL16-I123A181 cDNA with a C terminal flag and His-tag was purchased from Vigene Biosciences (Jinan, Shandong Province, China). Plasmids encoding CXCL16-I123V181, T123A181, and T123V181 cDNA mutations were generated by site-directed mutagenesis (Stratagene, La Jolla, CA, United States). The primers for 123T were 5′-TGAGGCCTGAGAAgTTGGGGGGCTGGTAGGAA-3′ (forward) and 5′-CAACTTCTCAGGCCTCAGAGGGGGCA-3′ (reverse). The primers for 181V were 5′-CCCAaCTGCC AGACTGTGGCCCGCA-3′ (forward) and 5′-ACAGTCTG GCAGtTGGGCCTGAGGCTGGGGA-3′ (reverse). The sequences of the four plasmids were checked by direct sequencing (BGI Genomics, Beijing, China). To obtain recombinant human CXCL16 proteins, CHO cells were cultured in a six-well plate (F12 medium with 10% FCS, penicillin-streptomycin, 3 mM glutamine). After washing was performed, CHO cells were cultured in serum-free medium and transfected with 4 μg of CXCL16-I123A181, I123V181, T123A181, or T123V181 plasmids. Transfected cells were cultured in serum-free medium for 48 h. According to the manufacturer’s instructions, the supernatants were subsequently purified by a His-bind purification kit (Merck-Millipore, Burlington, MA, United States) to obtain purified CXCL16-I123A181, I123V181, T123A181, and T123V181 proteins.

### Cell Chemotaxis and Adhesion Assay

The polycarbonate membrane in the middle of the Transwell insert (Corning, New York, United States) divides the chamber into two parts: the upper chamber and the lower chamber. THP-1 cells (human monocyte cell strain) were cultured in the upper chamber of the Transwell insert. The purified human CXCL16-I123A181, I123V181, T123A181, and T123V181 proteins were dissolved in the medium of the lower chamber (100 ng/ml). Chemotactic cells that passed through the membrane pores were dyed and subsequently counted to calculate the chemotaxis ability of four kinds of human CXCL16 protein (Figure 2B).

**FIGURE 1:**
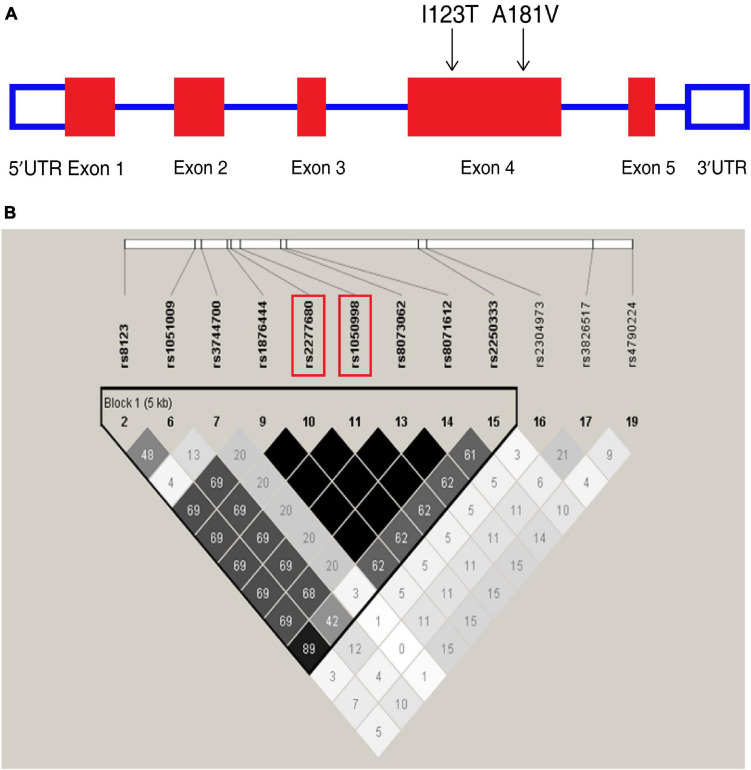
Overview of tagging single nucleotide polymorphisms (tag SNPs) in the CXCL16 gene. **(A)** CXCL16 gene organization and the location of two missense mutations, I123T (rs1050998) and A181V (rs2277680), on chromosome 17. **(B)** Location of SNPs in the CXCL16 gene with a minor allele frequency ≥5%. A linkage disequilibrium (LD) plot of these SNPs is displayed by a color scheme. Black represents very high LD (*r*^2^ = 1.0), and white indicates the absence of correlation (*r*^2^ = 0) between two SNPs. The *r*^2^ between I123T and A181V is 1.0.

**FIGURE 2:**
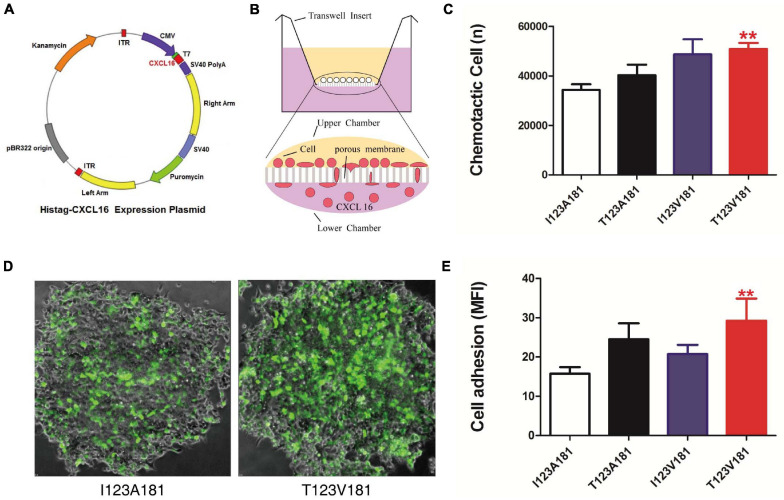
T123V181 haplotype enhances the chemotaxis and adhesion of inflammatory cells. **(A)** Map of the CXCL16 expression plasmid. **(B)** Schematic diagram of the chemotactic assay. The percentage of THP-1 cells recruited to the lower chamber was calculated to represent chemotaxis. **(C)** The chemotaxis of the CXCL16-T123V181 protein was significantly higher than that of the other three proteins (**P < 0.01). **(D)** The percentage of THP-1 cells with green fluorescence expressing transmembrane forms of CXCL16-T123V181 protein attached to RAW264.7 cells was calculated to represent the adhesion ability. **(E)** The adhesion ability of cells expressing CXCL16-T123V181 protein was significantly higher than that of the other three proteins (**P < 0.01).

For the cell adhesion test, THP-1 cells were cultured in serum-free medium and transfected with 4 μg of CXCL16-GFP-I123A181, I123V181, T123A181, and T123V181 expression plasmids. Raw264.7 cells that express CXCL16 receptor- CXC chemokine receptor 6 (CXCR6) were seeded at a density of 1 × 10^4^ in a six-well plate (Li et al., 2012). Raw264.7 cells were cultured for 48 h until they reached 70–80% confluency and were subsequently co-cultured with THP-1 cells expressing CXCL16-GFP-I123A181, I123V181, T123A181, and T123V181. The plate was incubated for 60 min at 37°C to enable cell binding. Non-adherent THP1 cells were washed away. The plate was read at 488 nm in a fluorescent plate reader. All assays were performed in triplicate. The number of THP-1 cells that adhered to Raw264.7 cells is presented as the mean fluorescence intensity (MFI).

### Molecular Modeling of CXCL16 Polymorphisms

Using MAFFT (v7.38)^1^, the CXCL16 proteins of 11 species were sequenced, and the evolutionary tree was calculated. The structurally conserved regions and loop regions of CXCL16 were analyzed by SMART^2^. The optimized structures were compared by PyMOL (version 0.97). Polymorphism Phenotyping v2 (Polyphen-2)^3^ was employed to predict the effect of the two mutations. Using Modeler (v10.1) (Webb and Sali, 2016), Phyre2 (v2.0) (Kelley et al., 2015) and RaptorX (Wang et al., 2016) with their default parameters, the full-length CXCL16 sequence was modeled. Using the wild-type CXCL16 structure as the template, the structure models of I123T and A181V are established. Using GROMACS 5.12, the structural models of I123T and A181V were modeled. The distances of amino acids and the area and volume of the hydrophobic pocket were analyzed using the distance geometry method.

### Statistical Analysis

The relevance of CXCL16 tag SNPs for clinical outcomes was analyzed by three genetic models, that is, the dominant, recessive, and allele-dose models. To assess sepsis risk, adjusting for age, sex, and injury severity for confounding effects, odds ratios with 95% confidence intervals were calculated by multivariable logistic regression models. Adjusted by age, sex, and injury severity for confounding effects, the association of tag SNPs with MOD scores was determined by linear regression analysis. A *P*-value < 0.05 after Bonferroni correction for multiple testing was employed to indicate significance. SPSS 13.0 software (SPSS Inc., United States) was utilized for the statistical analysis. Chemotaxis and adhesion data were compared using One-way analysis of variance (One-way ANOVA).

## Results

### CXCL16 I123T and A181V Are Two Tag SNPs That Are in Strong Linkage Disequilibrium

Chemokine C-X-C motif ligand 16 is located on chromosome 17 (Figure 1A). In the Chinese Han cohort, 20 SNPs were found in CXCL16. Among these SNPs, 12 had minor allele frequencies (MAFs) greater than or equal to 5%. Twelve SNPs constructed one haplotype block. Based on the analysis of the tagging threshold of *r*^2^, rs1050998 (I123T) and rs2277680 (A181V) were selected as tag SNPs. Also, I123T and A181V are located in exon 4, which encodes the mucin-like stalk domain of CXCL16. According to the genotyping data of 45 Han Chinese from the HapMap database, the two missense mutations are in strong linkage disequilibrium (D′ = 1; *r*^2^ = 0.99) (Figure 1B). Furthermore, I123T and A181V were genotyped in the three cohorts’ blood samples using the iMLDR method (Gan et al., 2019).

Major trauma patients from Chongqing (*n* = 920), Zhejiang (*n* = 350) and Guiyang (*n* = 350) were recruited for this study (Table 1). Both I123T and A181V were determined to be in Hardy-Weinberg equilibrium (HWE) among the three cohorts. Four haplotypes and their frequencies of the three cohorts are shown in Table 2. Notably, I123A181 and T123V181 are the two most common haplotypes out of four possible, and they are in strong linkage equilibrium (D′ = 1; *r*^2^ = 0.99 in both cohorts). This result means that there is almost no recombination between the two SNPs and no recombination in the haplotype region (Daly et al., 2001).

**TABLE 1:** Overall clinical characteristics of patients with major trauma.

	**Screening cohort**	**Validation cohorts**	
	**Chongqing (*N* = 920)**	**Zhejiang (*N* = 350)**	**Guizhou (*N* = 350)**
Age (years)	43.5 ± 15.1 (18-65)	42.5 ± 12.0(19–62)	37.6 ± 12.5 (18–65)
Male/female, n	681/239	268/82	273/77
**Injured body regions, n (%)**			
Head, n	475(51.6)	224 (64.0)	207 (59.1)
Thorax, n	536(58.2)	216 (61.7)	198 (56.6)
Abdomen, n	381(41.4)	129 (36.9)	116 (33.1)
Extremities, n	416(45.2)	198 (56.6)	187 (53.4)
**Number of regions injured, n (%)**			
One, n	461 (50.1)	151(43.1)	146(41.7)
Two, n	292 (31.7)	132 (37.7)	129 (36.9)
Three or above, n	167 (18.2)	67 (19.1)	75 (21.4)
ISS	23.4 ± 9.8	22.4 ± 8.1	21.5 ± 9.1
≥16, <25, n (%)	567 (61.6)	201 (57.4)	221(63.1)
≥25, n (%)	353 (38.7)	149 (42.6)	129(36.9)
**Organ dysfunction, n (%)**			
**None, n**			
One, n	281 (32.2)	112 (32.9)	125 (34.1)
Two, n	121 (14.5)	56(16.5)	51(13.9)
Three or above, n	41 (4.9)	23(6.8)	35(9.5)
Sepsis, n (%)	347 (37.7)	118 (33.7)	132(37.7)
**Source of infection, %**			
Respiratory tract infection	42.3	40.5	42.8
Primary bloodstream infection	22.1	22.9	20.3
Urinary tract infection	15.0	12.5	13.1
Catheter associated infection	10.6	8.2	9.5
Wound infection	7.3	8.8	10.2
Others*	2.7	7.1	4.1

**TABLE 2:** Distribution of haplotypes of CXCL16 I123T and A181V among trauma patients in three cohorts.

**Study Cohort**	**I123A181 (n, %)**	**I123V181 (n, %)**	**T123A181 (n, %)**	**T123V181 (n, %)**
Chongqing	548 (59.57)	3 (0.33)	1 (0.11)	368 (40.0)
Zhejiang	182 (52.0)	0 (0)	1 (0.29)	167 (47.71)
Guizhou	176 (50.29)	2 (0.57)	0 (0)	172 (49.14)

### T123V181 Haplotype Is Associated With an Increased Risk of Sepsis and MODS in Major Trauma Patients

As I123T and A181V were determined to be located in one haplotype block and observed to be in strong linkage equilibrium, we further compared their haplotype frequencies according to sepsis morbidity rate and MOD scores among major trauma patients from Chongqing, Zhejiang and Guizhou cohorts. There were no significant differences in gender, age or injury severity score among patients in the three cohorts. Among the four haplotypes, T123V181 was found to be associated with an increased risk for sepsis morbidity in both cohorts (OR = 1.89, 95% CI = 1.82–2.56, and *P* = 0.001 for the Chongqing cohort; OR = 1.76, 95% CI = 1.53–2.18, and *P* = 0.004 for the Zhejiang cohort; OR = 1.55, 95% CI = 1.42–1.96 and *P* = 0.012 for the Guizhou cohort, Table 3). Notably, T123V181 carriers also were observed to have significantly higher MOD scores than I123A181 carriers among major trauma patients in the three cohorts (*P* = 0. 0016 for the Chongqing cohort; *P* = 0.002 for the Zhejiang cohort; *P* = 0.001 for the Guizhou cohort, Table 3).

**TABLE 3:** Haplotype effects of the I123T and A181V polymorphisms on the incidence of sepsis among trauma patients in three cohorts.

	**Haplotypes**	**N**	**Age(yr)**	**Sex(M/F)**	**ISS**	**Sepsis, n/**	**MOD score**
Chongqing	I123A181	548	44.2 ± 12.9	379/136	22.6 ± 9.3	181(33.0%)^ a1^	6.3 ± 2.5^b1^ 5
	I123V181	3	43.5 ± 13.7	2/1	21.5 ± 5.7	0(%)	6.6 ± 1.3
	T123A181	1	48.0	1/0	20.0	0(%)	7
	T123V181	368	43.7 ± 13.3	299/102	21.6 ± 9.4	166(45.1%)	7.8 ± 2.7
Zhejiang	I123A181	182	42.6 ± 11.8	138/44	22.3 ± 7.1	49(26.9%)^ a2^	6.7 ± 3.0^b2^
	I123V181	0	–	–	–	–	–
	T123A181	1	46	1/0	22.0	1(100%)	7.0
	T123V181	167	43.5 ± 14.7	129/38	19.7 ± 7.5	68(40.7%)	8.1 ± 2.9
Guizhou	I123A181	176	37.4 ± 12.5	141/42	22.8 ± 9.2	45(25.6%)^ a3^	6.9 ± 2.5^b3^
	I123V181	2	38.5 ± 12.0	2/0	21.5 ± 3.0	0(%)	7.5 ± 2.6
	T123A181	0	–	–	–	–	–
	T123V181	172	39.8 ± 11.9	130/35	22.6 ± 8.9	87(50.6%)	8.5 ± 2.9

*a: dominant effect (variant homozygotes +heterozygotes vs. wild homozygotes) as analyzed by ANCOVA,^ a1^P = 0.001, ^*a*2^P = 0.004, ^*a*3^P = 0.012. b: recessive effect (variant homozygotes vs. heterozygotes + wild homozygotes) as analyzed by ANCOVA, ^*b1*^P = 0.0016, ^*b2*^P = 0.002,^ b3^P = 0.001.*

### T123V181 Haplotype Enhances the Chemotaxis and Adhesion of Human Monocytes

During sepsis, innate immune cells in peripheral blood, such as neutrophils, monocytes and macrophages adhere to endothelial cells and then reach the infection site through endothelial cells and matrix layer. The migration of inflammatory cells facilitates the clearance of bacteria, but the migration and aggregation of a large number of inflammatory cells can also lead to sepsis through the release of uncontrollable cytokines and inflammatory response. Multiple organ dysfunction is mainly caused by excessive recruitment of innate immune cells migrate to non-inflammatory sites. Considering that CXCL16 is a crucial chemokine, we speculated that the amino acid changes of CXCL16 protein induced by I123A181 haplotypes might influence the chemotaxis and adhesion function of innate immune cells expressing CXCL16 protein, and thus cause the difference of the sepsis morbidity rate. The two missense mutations rs1050998 (I123T) and rs2277680 (A181V) confer T→C and G→A substitutions at positions 4585486 and 4585312 on chromosome 17, leading to a substitution of isoleucine to threonine at codon 123 (I123T) and alanine to valine at codon 181 (A181V) at opposing ends of the mucin-type stalk region (Petit et al., 2011). Thus, we further elucidated the effect of four haplotypes of the two missense mutations on the chemotaxis and adhesion of human monocytes. Expression plasmids encoding human CXCL16-I123A181, I123V181, T123A181, and T123V181 cDNA were transfected into CHO cells to obtain purified soluble CXCL16 proteins (Figures 2A,B). The results of this experiment showed that significantly more THP-1 cells migrated to the low chamber when the chemotactic protein in the low chamber was CXCL16-T123V181 (*P* < 0.01) (Figure 2C). To verify the cell adhesion ability caused by the missense mutation, THP-1 cells transfected with CXCL16-GFP-I123A181, I123V181, T123A181, and T123V181 expression plasmids were co-cultured with RAW264.7 cells that expressed CXCL16 receptor-CXCR6. After co-culture for 48 h, non-adherent THP-1 cells were washed away, and the fluorescence intensity showed the cell adhesion ability of transmembrane forms of CXCL16. The results showed that significantly more THP-1 cells with green fluorescence expressing CXCL16-T123V181 protein were attached to RAW264.7 cells (Figures 2D,E).

### Conservation and Protein Structure Analysis of CXCL16

**FIGURE 3:**
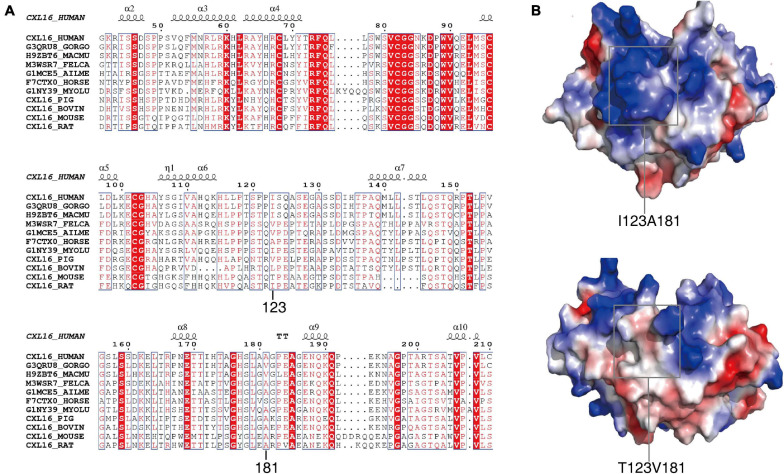
Conservative analysis and molecular modeling of I123T and A181V. **(A)** Conservative analysis shows that the two missense mutations are both located in the non-conserved region of the CXCL16 gene. **(B)** The structural diagram shows that the active center of the CXCL16 protein was changed by V → A substitution, which might make the active center of the CXCL16 protein smaller.

The 123 and 181 amino acids of the CXCL16 protein are not highly conserved in 11 mammalian species and possess a high probability of protrusion. Both amino acids 123 and 181 may be located in non-conserved regions (Figure 3A). Polymorphism Phenotyping v2 (Polyphen-2, see text footnote 3) was employed to predict the effect of the two mutations. The results showed that the effect of the two mutations on the protein structure was BEGIEN, which meant that the two amino acid mutations would not have a destructive effect on the protein structure. Molecular modeling analysis further indicated that I123T was located on the surface of CXCL16, which might have only a slight effect on the structure of the native protein. A181V is located in the interior of the protein, which had a strong effect on the structure of the CXCL16 protein (Figure 3B). The results of structure comparison also showed that the average root mean squared deviation (RMSD) of I123T was only 1.62, while the average RMSD of the mutation region of A181V was 1.95, which meant that the structural change of the mutation region of A181V was greater. The structural diagram showed that the active center of the CXCL16 protein might have been changed. The V → A substitution was more hydrophobic, which might reduce the size of the active center of the CXCL16 protein (Figure 3B).

## Discussion

A variety of molecules mediate immunocyte chemotaxis and adhesion. Intercellular adhesion molecule-1 (ICAM-1) is a transmembrane glycoprotein, which is usually expressed in endothelial cells and immune cells and plays a key role in leukocyte migration and activation. Vascular cell adhesion molecule-1 (VCAM-1) mediates a variety of innate immune cells to adhere to the vascular endothelium, and plays a role in leukocyte endothelial cell signal transduction. Selectin is only expressed in activated vascular endothelial cells and mediates the initial adhesion and interaction between leukocytes and vascular endothelial cells. ICAM-1, VCAM-1 and Selectins are crucial adhesion molecules that mediate the adhesion

between endothelial cells and immune cells. However, CXC chemokines are produced locally and initiate innate immune cells recruitment during inflammation. CXCL16 is a microbially regulated chemokine which plays a regulatory role in innate immunity by attracting CXC chemokine receptor 6 (CXCR6) expressing cells (Abel et al., 2004; Olszak et al., 2012). CXCL16 and CXCR6 were recently observed to be related to various inflammatory diseases, such as glomerulonephritis (Garcia et al., 2007), pulmonary diseases (Zuo et al., 2019), atherosclerosis (Aslanian and Charo, 2006), coronary artery disease (Zhou et al., 2016), rheumatoid arthritis (van Lieshout et al., 2009) and many inflammation-related cancers (Kee et al., 2014; Singh et al., 2016; Chung et al., 2017). At present, there are only four studies investigating the clinical associations between CXCL16 polymorphisms and atherosclerosis (Wang et al., 2010; Zivković et al., 2015), coronary heart disease (Petit et al., 2011) and multiple sclerosis (Stojković et al., 2014). The protein structure of CXCL16 consists of four domains, and the 123rd and 181st amino acids are located in glycosylated mucin-like domains. The chemotaxis function of CXCL16 is primarily mediated by the chemokine domain which can bind to CXCR6. The glycosylated mucin-like domain can affect the conformation of the chemotaxis domain (Petit et al., 2008). The missense mutation of 123rd and 181st amino acids may influence the function of innate immune cells which express CXCL16 by altering the structure of glycosylated mucin like domains. To date, there is little information regarding the clinical relevance of CXCL16 polymorphisms in trauma patients with sepsis and MODS.

Case-control studies are commonly utilized association studies employed to identify the genetic basis of diseases. One of the main limitations of this approach is inappropriate case-control matching, such as the use of healthy blood donors as a control group, which leads to population stratification. Therefore, we only chose trauma patients and followed them prospectively to determine whether those with genetic variation had different risks of posttraumatic sepsis and MODS. In this research, we investigated the potential clinical relevance of two missense SNPs of CXCL16, namely, I123T (rs1050998) and A181V (rs2277680). Our results indicated that among the four haplotypes of I123T and A181V, T123V181 was associated with an increased risk for sepsis morbidity rate and a higher MOD score in the Chongqing population. This clinical relevance of T123V181 was further confirmed in another two independent cohorts, the Zhejiang and Guizhou cohorts.

The structure of CXCL16 is composed of a chemokine domain, a mucin like stalk, a transmembrane domain and cytoplasmic tail. This structure suggests that CXCL16 potentially functions as an adhesion molecule and a soluble chemoattractant once cleaved from the cell surface (Wang et al., 2016). Thus we further elucidated the influence of the four haplotypes of CXCL16 on the adhesion and chemotaxis ability of innate immune cells. *In vitro* chemotactic experiments showed that the CXCL16-T123V181 protein enhanced the chemotaxis ability of monocyte. The adhesion ability of THP-1 cells expressing T123V181 to immunocytes was also stronger than that of the other three haplotypes. During sepsis, a large number of inflammatory cells migrate and aggregate and cause organ dysfunction by releasing excessive cytokines and over-activating immune response. The amino acid changes induced by CXCL16-T123V181 haplotype can enhance the chemotaxis and adhesive ability of CXCL16 protein on innate immune cells. Excessive chemotaxis and adhesion of innate immune cells may be the cellular mechanism of patients who carry CXCL16-T123V181 haplotype has the tendency to have sepsis after major trauma.

We further analyzed the structural changes of CXCL16-T123V181 protein, because this might be the molecular mechanism of enhanced migration and adhesion ability of innate immune cells in CXCL16-T123V181 carriers. Conservation and protein structure analysis of CXCL16 showed that the average RMSD of the mutation region of A181V was 1.95, which meant that the structural change of the mutation region of A181V was greater. The morbidity of posttraumatic sepsis and the MODS score in T123V181 carriers were higher than those in I123A181 carriers, which might be observed because of the structural change caused by CXCL16-T123V181. The structural diagram showed that the active center of the CXCL16 protein might have changed, and the V → C substitution was more hydrophobic, which might reduce the size of the active center of the CXCL16 protein. Notably, T123 and V181 changed the structure of the CXCL16 protein active center, which led to changes in protein function and changes in adhesion and chemotaxis of CXCL16-expressing immunocytes and, finally, led to changes in posttraumatic sepsis morbidity and the MODS score.

It must be noted that our research has several limitations. The sample size of Guizhou and Zhejiang populations is relatively small compared with Chongqing populations. Second, it is difficult to obtain additional blood samples to detect the serum level of CXCL16 in patients with sepsis. Third, only patients of the Han nationality were recruited in this study. The Han nationality is the most populous ethnic group in China, and a biologically relevant phenotype and an ethnically consistent population might maximize the possibility of finding meaningful genetic associations. Although the number of patients from Zhejiang and Guizhou populations is relatively small to test the impact of SNPs on immunocyte function and clinical trajectory, the correlation between the I123A181 haplotype and sepsis in the Chongqing population was verified in the Zhejiang and Guizhou populations, and these findings can be extrapolated to other populations. Effective targeted therapy for sepsis requires an understanding of the heterogeneity of the individual host response. A precise approach using biomarkers enables the identification of subgroups of patients with different host responses and specific individuals who are likely to benefit from personalized therapy. Our genetic approach may employ genomic information to classify individuals into subpopulations that differ in their susceptibility to sepsis, in the pathophysiology of sepsis, and in their response to a specific treatment according. Therefore, our results defined the functional significance of the I123A181 haplotype of CXCl16 for the first time and demonstrated that it might be employed as a biomarker for sepsis and MODS in severe trauma patients. Our genetic methods can be employed to classify individuals and their susceptibility to sepsis and MODS in different populations according to their specific genetic information.

## Conclusion

We investigated the clinical relevance of two missense SNPs, I123T and A181V, in CXCL16. Notably, T123V181 haplotypes were observed to be associated with an increased risk for sepsis morbidity rate and higher MOD score in three independent cohorts. *In vitro* chemotactic and adhesion experiments showed that T123V181-CXCL16 enhanced the chemotaxis and adhesion ability of immunocytes. Conservation and protein structure analysis of CXCL16 showed that T123 and V181 changed the structure of the CXCL16 protein active center, possibly leading to changes in protein function and changes in the adhesion and chemotaxis of CXCL16-expressing immunocytes. Our findings provide insight into CXCL16 T123V181 haplotypes as a novel biomarker for improving the early identification of high risk for traumatic sepsis or MODS.

## Data Availability Statement

The original contributions presented in the study are included in the article/supplementary material, further inquiries can be directed to the corresponding author/s.

## Ethics Statement

The studies involving human participants were reviewed and approved by the Ethics and Protocol Review Committees of Army Medical University. The patients/participants provided their written informed consent to participate in this study.

## Author Contributions

JS and HZ were the main researchers in this study. DL, LC, QW, LG, and DW were involved in the collection of blood samples and clinical data. JW, JuD, HH, and AZ performed the technical work. JiD, JJ, and LZ planned the study. LZ wrote the manuscript. All authors read and approved the final manuscript.

## Conflict of Interest

The authors declare that the research was conducted in the absence of any commercial or financial relationships that could be construed as a potential conflict of interest.

## Publisher’s Note

All claims expressed in this article are solely those of the authors and do not necessarily represent those of their affiliated organizations, or those of the publisher, the editors and the reviewers. Any product that may be evaluated in this article, or claim that may be made by its manufacturer, is not guaranteed or endorsed by the publisher.

## Footnotes

1
https://mafft.cbrc.jp/alignment/software/algorithms/algorithms.html

2
https://www.megasoftware.net/

3
http://genetics.bwh.harvard.edu/pph2/
